# Ma Xing Shi Gan Decoction Protects against PM2.5-Induced Lung Injury through Suppression of Epithelial-to-Mesenchymal Transition (EMT) and Epithelial Barrier Disruption

**DOI:** 10.1155/2020/7176589

**Published:** 2020-06-17

**Authors:** Ye-fang Wang, Yu-xiang Fei, Bo Zhao, Qi-yang Yin, Jian-ping Zhu, Guang-hui Ren, Bo-wen Wang, Wei-rong Fang, Yun-man Li

**Affiliations:** ^1^Department of Paediatrics, Nanjing Integrated Traditional Chinese and Western Medicine Hospital, Nanjing 210014, China; ^2^State Key Laboratory of Natural Medicines, School of Basic Medical Sciences and Clinical Pharmacy, China Pharmaceutical University, Nanjing 210009, China

## Abstract

This research was designed to explore the effect of Ma Xing Shi Gan decoction (MXD) in alleviating particulate matter less than 2.5 *μ*m in diameter (PM2.5) induced lung injury from the perspective of epithelial barrier protection and inhibition of epithelial-to-mesenchymal transition (EMT). Rats were exposed to PM2.5 to establish a lung injury model in vivo, and a PM2.5-stimulated primary cultured type II alveolar epithelial cell model was introduced in vitro. Our results indicated that MXD alleviated the weight loss and pathologic changes and improved the epithelial barrier dysfunction. MXD also significantly inhibited the TGF-*β*/Smad3 pathway, increased the level of ZO-1 and claudin-5, and reversed the EMT process. Notably, the protection of MXD was abolished by TGF-*β* in vitro. Our results indicated that MXD has a protection against PM2.5-induced lung injury. The proposed mechanism is reversing PM2.5-induced EMT through inhibiting TGF-*β*/Smad3 pathway and then upregulating the expression of tight-junction proteins.

## 1. Introduction

Particulate matter (PM) is a major component of air contamination. There is a positive correlation between PM concentrations and the morbidity (and mortality) rates, which was evidenced by epidemiologic studies [1–4].

PMs less than 2.5 *μ*m in diameter (PM2.5) are likely to be deposited deep in the lungs [5]. Recent studies have testified that PMs contribute to airway hyperactivity, macrophage excessive activation, and epithelial damage [6–10].

Clinically, there are no specific medicines for treating pulmonary diseases related to PM2.5 exposure, facilitating the discovery of novel, safe, and effective agents. Ma Xing Shi Gan decoction (MXD), a centuried classical decoction consisting of ma-huang, ku-xing-ren, gan-cao, and shi-gao has been used for the treatment of disease of the respiratory system including cough, bronchial inflammation, pneumonia, and asthma for more than 2000 years [11, 12].

As a critical line of defence, the airway epithelium regularly forms a barrier against invasion of inhaled environmental agents including but not limited to pollutants and pathogens [13]. The epithelial barrier function largely depends on the tight junction surrounding epithelial cells, which forms ordered belt-like structure and regulate the flow of foreign substances [14]. In response to epithelial challenge (including particulate, radiation, drug stimulation, bacterial/virus infection), epithelial cells will undergo epithelial-to-mesenchymal transition (EMT), contributing to the resultant fibrosis [15, 16]. Research studies have indicated that PM2.5 can initiate the EMT of epithelial cells [17–19]. A representative event during EMT process is the decomposition of tight junctions, which is evidenced by the redistribution of barrier-related proteins including zonula occluden 1 (ZO-1) and claudin-5, concomitant with loss of the cell polarity and acquisition of fibroblast-like phenotype. However, it remains unclear whether MXD can inhibit PM2.5-induced EMT, improve the epithelial barrier function, and thus attenuate lung injury.

This research was aimed at evaluating the protection of MXD in attenuating lung barrier dysfunction induced by PM2.5, exploring if the effect is mediated by alleviating TGF-*β*/Smad-dependent EMT progress, and thus improving the barrier function of epithelial cell.

## 2. Materials and Methods

### 2.1. Reagents

*Ephedrae herba* (ma-huang) was purchased from Sanyue Traditional Chinese Medicine Co., Ltd. *Armeniacae amarum semen* (ku-xing-ren) was purchased from Jiangsu Huahong Pharmaceutical Technology Co., Ltd. *Glycyrrhizae radix preparata* (gan-cao) was purchased from Hangzhou Zhende Traditional Chinese Medicine Co., Ltd. *Gypsum fibrosum* (shi-gao) was provided by Jiangsu Huahong Pharmaceutical Technology Co., Ltd. Botanical identifications were authenticated by the Department of Pharmacology of Chinese Medicine (China Pharmaceutical University, Nanjing, China). The quality control of herb drugs is consistent with rule of related part of Chinese Pharmacopoeia (2015 edition). Voucher specimens of *Ephedrae herba*, *Armeniacae amarum semen*, *Glycyrrhizae radix preparata*, and *Gypsum fibrosum* have been deposited in the specimen room of Nanjing Integrated Traditional Chinese and Western Medicine Hospital and registered under the numbers No-1518M4, No-15180K3, No-15180G2, and No-1518S24, respectively.

PM2.5 samples collected from the city of Nanjing (China) were donated by the Environmental Monitoring Organization of Nanjing. The PM2.5 sample was suspended in ultrapure water and dispersed with an ultrasonic washer at 40 kHz for 1.5 h. After filtered by an 8-fold gauze, the suspension was centrifugated at 3000*g* for 15 min to remove the supernatant. The deposit was freeze-dried and ultraviolet sterilized for 1 h, after which the depurated PM2.5 was obtained.

All other reagents (analytically pure) could be purchased through routine channel.

### 2.2. Preparation and Analysis of MXD

*Ephedrae herba* (4 g) was decocted in ultrapure water (1000 mL) for 60 min. After discarding the float foam, *Armeniacae amarum semen*, *Glycyrrhizae radix preparata*, and *Gypsum fibrosum* (12 g: 8 g: 24 g) were added in and boiled under a reflux condenser for another 40 min. The dregs were removed by filtration, and the extract was enriched to 2 g/mL by rotary evaporation (in terms of the weight of crude drugs). Qualitative and quantitative determination of the main components of MXD by UPLC-MS/MS was performed using an external standard method just as our recently published article (a total of 1.4 L MXD was prepared, uniformly mixed, and divided into individual brown reagent bottles, and the same batch of MXD was used in all the experiments) [20]. Information of the four herbs is shown in Table 1.

### 2.3. Animals and Drug Administration

SPF male Sprague-Dawley rats (180–200 g, 5-6 weeks) were purchased from the Qinglongshan Animal Cultivation Farm (Nanjing, China), and 20 SPF ICR mice (21–25 g, half male and half female, only for safety evaluation) were purchased from the Comparative Medical Centre of Yangzhou University. All animals were fed with standard complete feed and purified water. All animals were kept at a relative humidity of 55 ± 5% (at 24 ± 2C) in compliance with institutional guidelines of China Pharmaceutical University and the National Institutes of Health guide for the care and use of laboratory animals (NIH Publications No. 8023, revised 1978). All experiments were approved by the Institutional Animal Care and Use Committee of China Pharmaceutical University (SYXK (Su) 2016-0011).

### 2.4. Experimental Design

After a habituation for one week, rats were randomly assigned in six groups: blank control group (sham-operated group), intervention group (16.4 g/kg), PM2.5 model group, PM2.5 + MXD (4.1, 8.2, and 16.4 g/kg) groups. The medium dose (8.2 g/kg) was 6.5 times of the dosage in clinical application, which was calculated according to the conversion coefficient between rat and human [40]. On clinical uses, MXD was administered for 5 to 10 days as a course of treatment. Hence, in this experiment, MXD was diluted with normal saline and administered intragastrically once a day for 7 consecutive days. Rats in the control group and PM2.5 group received the same amount of normal saline instead of MXD. The required number of rats is listed in Table 2 as follows:

The same animals were used for determination of clinical signs, body weight, lung edema, hematoxylin and eosin (H&E) staining, immunohistochemical (IHC) assay, enzyme-linked immunosorbent assay (ELISA), and western blot. Another 36 rats were used for the collection of bronchoalveolar lavage fluid (BALF) collection.

The safety of MXD was evaluated in mice and rats (Supplementary Figure 1).

### 2.5. Establishment of Acute Lung Injury Model

After anesthetization with 3% isoflurane, rats were fixed at supine position. PM2.5 challenge was introduced by intratracheal instillation. Briefly, a polyethylene tube (0.6 mm in diameter) was inserted into trachea, and then PM2.5 suspension (0.1 mL/100 g) was instilled. The dosage of PM2.5 was based on respiratory parameters of rats and the air-quality reports of Nanjing [41]. The average tidal volume of rats (200 g) is 0.86 mL, and the respiratory rate is 5100 times/h. The total respiratory volume for one day is 0.105 m^3^. Based on the annual average PM2.5 concentration (43 *μ*g/m^3^) in Nanjing, the daily exposure of PM2.5 is 4.515 *μ*g. The modified PM2.5 exposure concentration was determined at 2.3 mg/kg body weight after multiplied by an uncertainty factor of 100-fold. The concentrations of 2.3 mg/kg, 4.6 mg/kg, and 9.2 mg/kg were chosen for preliminary model evaluation. In case of agglomeration, PM2.5 was well-diversified in ultrapure water by ultrasound concussion before instillation. The intratracheal instillation was performed at the 1st, 3rd, 5^th^, and 7th days. Based on the result of preliminary experiment, the dose of 9.2 mg/kg was selected for establishing lung injury. MXD was administered 1 h after the last PM2.5 exposure. To date, there is a lack of clinical treatments for PM2.5-induced lung injury, so patients with similar pulmonary disease are usually treated with antibiotics. However, based on our preliminary experiment, subcutaneous injection of clarithromycin or penicillin showed no therapeutic effects in alleviating the lung injury, which may be attributed to the relatively sterile experimental environment (data are not shown). Hence, we didn't set a positive control group in the animal experiment. The successful establishment of lung fibrosis was evidenced by the Masson staining and the expression of Collagen I and III (Supplementary Figure 2).

### 2.6. Assessment of Body Weight and Clinical Signs in Rats

Rats were weighed before and after receiving PM2.5 exposure (13 consecutive days). The clinical scoring system including weight loss, piloerection, rapid shallow breathing, and lethargy were assessed, and each symptom was assigned one point [42].

### 2.7. Lung Water Content Assay

Lung edema was estimated by the wet/dry method. The left lung was weighed for obtaining the wet weight, followed by desiccation in a drying oven (80°C) for 48 h. Then, the dry weight was obtained by weighing again. The water containing (%) in the lung was determined as follows:

(1) $$\begin{array}{c} \text{water containing}=\frac{\text{wet weight }-\text{dry weight}}{\text{lung wet weight }}\times 100\text{\%}.\; \end{array}$$

### 2.8. Histopathological Evaluation of the Lung Tissue

After isolating 0.4 g right lung tissue for homogenate preparation and protein extraction, the rest of right lung tissues were fixed in 10% formaldehyde for 48 h, followed by dehydration in graded ethanol and embedding in paraffin. Finally, the tissues were sliced into sections with 5 *μ*m in thickness and then H&E staining was conducted. Pathologic examination was assessed using a light microscope (Olympus, Japan, BX53). Histological characteristic was scored 0–5 as described previously [43].

### 2.9. IHC Assay

The alternation in the level of E-cadherin (a classical epithelial marker) and vimentin (a fibroblastic marker) during lung injury was studied by the IHC assay. The 5-*μ*m slices were deparaffinized in xylene, hydrated by graded alcohol. To retrieve the antigen, slices were placed in an autoclave (10 min, 121°C). The endogenous peroxidase activity was blocked by incubating slices in 3% H_2_O_2_ at 25°C for 0.5 h. Sections were washed with PBS (5 min × 3 times), and sections were incubated with 10% goat serum for 0.5 h at 37°C, followed by incubation with the primary antibodies against E-cadherin (CST, USA; 1 : 500) and vimentin (Wanleibio, China; 1 : 500) for 15 h at 4°C. Then, the sections were stained with the peroxidase-conjugated secondary antibody at 37°C for another 60 min. Immunostaining was then visualized using 3, 3′-diaminobenzidine and counterstained with hematoxylin.

### 2.10. Alveolar Barrier Damage

The alveolar barrier leakage was analysed by measuring total protein concentration and the number of infiltrated cells in the BALF. BALF was collected 2 h after the last administration. Briefly, a sterile polyethylene cannula was installed into trachea, after which a total of 2.0 mL normal saline was slowly instilled and collected by gravity. Finally, the total cell count in BALF was calculated. The BALF was centrifuged for 15 min at 1000 g to obtain the supernatant, and the total protein concentration in which was analysed with a bicinchoninic acid (BCA) kit (Jiancheng Bioengineering Institute, Nanjing, China).

### 2.11. Measurement of TGF-*β*1 in Lung Homogenates

0.2 g lung tissue was homogenized in 2 mL normal saline, followed by centrifugation at 1000*g* for a period of 15 min to obtain the supernatant. Then, the TGF-*β*1 level was determined by a corresponding ELISA kit (Tianjin Anoric Biotechnology Co., Ltd).

### 2.12. Primary Cell Culture

Primary type II “epithelial cells” were isolated from SPF male rats (180–200 g) as described previously with slight modification [44, 45]. Briefly, the rats were injected intraperitoneally with 750 U heparin and anesthetized (0.3% pentobarbital sodium, 1 mL/100 g) followed by endotracheal intubation. Then, the pulmonary circulation was perfused with normal saline to exclude blood. The lungs were lavaged 10 times with D-Hanks solution containing 0.2 mM EGTA to remove macrophages. The lungs were lavaged with 20 mL of porcine pancreatic elastase (Shanghai Yuanye Bio-Technology Co., Ltd, Shanghai, China) at 4.5 U/mL in D-Hanks solution, after which elastase solution (4 U/mL) was infused into total lung (about 10 mL). The lung was incubated in D-Hanks solution at 37°C. Then, the lungs were minced into pieces (1 mm^3^), and 20 mL D-Hanks solution supplemented with 20% FBS (Gibco) was added in for further inhibiting enzymatic activity. After stirred for 5 min (in a 37°C water bath at 130 cycle/min), the cell suspension was filtered through three filters (300 *μ*m, 100 *μ*m, and 20 *μ*m filter nylon mesh) to exclude debris of tissue and clumps of cells. The cells in the filtrate were suspended in DMEM (Gibco) and seeded into a culture flask with rat IgG (Solarbio Co., Ltd., Beijing, China), to remove cells bearing Fc receptors [45]. After a 1-hour incubation, the free cells were primarily type II alveolar epithelial cells (this step was repeated once). Cells were then cultured in DMEM supplemented with 15% FBS at 3 × 10^5^ cells/cm^2^ in 96-well culture plates (for cell viability assay) and 6-well culture plates (for morphological observation and western blot) and incubated in a cell incubator (37°C, 5% CO_2_ and 95% air). The primary alveolar type II cell was identified by immunocytochemistry staining of pulmonary surfactant-associated protein (Supplementary Figure 3).

### 2.13. Preparation of MXD-Medicated Serum

As an aqueous herbal extraction, MXD is a complex dispersion system containing small molecule alkaloids, polysaccharide, gelatinoid, aggregation, and precipitates, which restrict its direct application in vitro [46, 47]. Therefore, the method of serum pharmacology was introduced. Twenty rats (weighing 180–200 g) were divided into two groups: blank control group and MXD intervention group. The rats were raised in the same condition as described in Section 2.3. The MXD intervention group was treated with MXD at 16.4 g/kg once daily for 7 days. Two hours after the last administration, blood was drawn from the abdominal aorta, followed by centrifugation at 3000*g* for 15 min to obtain MXD-medicated serum. The serum from the same group was pooled, inactivated at 56°C for 30 min, filtered with filters (0.22 *µ*m in bore diameter) and frozen at −80°C for further use. Rats in the control group were administrated intragastrically with 0.9% NaCl in the same protocol, after which preparation of nonimmune serum was conducted (nonimmune serum was used to dilute the medicated serum for uniformity).

### 2.14. Cytotoxicity Assay

Optimal concentration selection of PM2.5 was evaluated with CCK8 kits (Solarbio, Beijing, China), and the concentration ranges of which were determined by referring to the published study [48]. Cells were stimulated with series concentrations of PM2.5 (5, 10, 15, 20, 25, 30, 35, and 40 *μ*g/cm^2^) for 48 h. Then, 20 *μ*L CCK8 solution was added, followed by an additional 2-hour incubation in an incubator at 37°C. The absorbance (450 nm) was determined with a microplate reader (Thermo scientific, USA, Multiskan FC).

Considering that the serum volume fraction (concentration) in a medium generally does not exceed 20%, the total serum volume fraction was strictly restricted to 20%. Cells were cultured in MXD-medicated serum at the following four concentrations (20% nonimmune serum; 5% medicated serum plus 15% nonimmune serum; 10% medicated serum plus 10% nonimmune serum; 20% medicated serum) for 48 h, followed by a cell viability assay to determine the nontoxic concentration range.

To analyse the protection of MXD-medicated serum against PM2.5-induced injury, rat primary cultured type II alveolar epithelial cells were grouped as follows: control group, PM2.5 group, and medication groups. Cells in the control group were treated with 20% nonimmune serum. Cells in the PM2.5 group and medication groups were exposed to PM2.5 (25 *μ*g/cm^2^). Cells in medication groups were treated with medicated serum at the volume fractions of 5%, 10%, and 20% (the volume fraction of serum was added to 20% using nonimmune serum for uniformity). The total serum volume fraction was strictly restricted to 20% for uniformity. Medicated serum was added simultaneously with PM2.5. After a 48-hour incubation, cell viability was determined using CCK8 kits.

### 2.15. Morphological Observation

After incubation with PM2.5 (25 *μ*g/cm^2^) and medicated serum (5%, 10% and 20%) for 48 h, the morphological changes induced by exposure of PM2.5 were observed under an inverted microscope at 400x magnification and photographed.

### 2.16. Measurement of TGF-*β*1 in Medium

After alveolar cells were incubated with PM2.5 (25 *μ*g/cm^2^) and medicated serum (5%, 10%, and 20%) for 48 h, the culture medium was collected, centrifugated at 3000*g* for 15 min to obtain the supernatant. The level of TGF-*β*1 in the supernatant was determined by ELISA kits (Tianjin Anoric Biotechnology Co., Ltd).

### 2.17. Western Blot

Primary alveolar cells were extracted, inoculated onto a 6-well plate and cultured for 24 h, after which cells were exposed to PM2.5 and the selected volume fractions of MXD-medicated serum. Cells were harvested for western blot after the 48-hour incubation.

Proteins in lung tissues and alveolar cells were extracted with radioimmunoprecipitation assay (RIPA) lysis buffer and quantified by a BCA kit. The proteins were separated on SDS polyacrylamide gel and transferred onto the polyvinylidene fluoride (PVDF) membrane (Merck Millipore Ltd.). After the blockage with 5% nonfat dry milk, the membrane was immersed into solutions containing specific primary antibodies: E-cadherin (CST, USA; dilution 1 :2000), vimentin (Wanleibio, Shenyang, China; dilution 1 :1000), ZO-1 (Bioss Antibodies, Beijing, China; dilution 1 :1000), occludin (Wanleibio, Shenyang, China; dilution 1 :1000), claudin-5 (Wanleibio, Shenyang, China; dilution 1 :1000), Smad3 (Wanleibio, Shenyang, China; dilution 1 :1000), and p-Smad3 (Abways, dilution 1 : 800) at 4°C overnight. The PVDF membranes were then washed with TBST, followed by incubation with the secondary antibody conjugated with HRP (Abways, Shanghai, China; dilution 1 : 5000) at room temperature for 1 h. Blots were washed with TBST and then developed with the enhanced chemiluminescence kit (Wanleibio, Shenyang, China). Semiquantitative analysis of the protein expression was performed using the ImageJ software (version 1.41).

### 2.18. Verification Experiment Using Recombinant TGF-*β* (rTGF-*β*)

To elucidate whether the protection of MXD was mediated by the inhibition of the TGF-*β*/Smad3 signal pathway, a verification experiment was conducted using rTGF-*β* (Neobioscience, Shanghai, China). Briefly, cells were grouped as follows: (1) control group; (2) PM2.5 group; (3) treatment group, cells challenged by PM2.5 exposure were incubated with 20% MXD-medicated serum; and (4) rTGF-*β* group, cells undergone PM2.5 stimulation were incubated with 20% MXD-medicated serum + rTGF-*β* (10 ng/mL). Medicated serum (or nonimmune serum) and rTGF-*β* were added simultaneously right after the stimulation of PM2.5. After a 48-hour incubation, cells were photographed and harvested. The expression of E-cadherin, *α*-SMA (Wanleibio, Shenyang, China; dilution 1 : 1000), vimentin, p-Smad3, ZO-1, and claudin-5 was semiquantitatively analysed.

### 2.19. Statistical Analysis

Data were visualized using GraphPad Prism 5.0. All statistical analyses were performed using SPSS 19.0 software and analysed by one-way analysis of variance (ANOVA) followed by the LSD post hoc test (variance homogeneity) to compare the difference between groups. When the variance was heterogeneous, Welch's ANOVA would be introduced followed by Games Howell test. A value of *P* < 0.05 was considered as statistically significant.

## 3. Results

### 3.1. MXD Mitigated Clinical Sign and Promoted Weight Recovery

**Figure 1:**
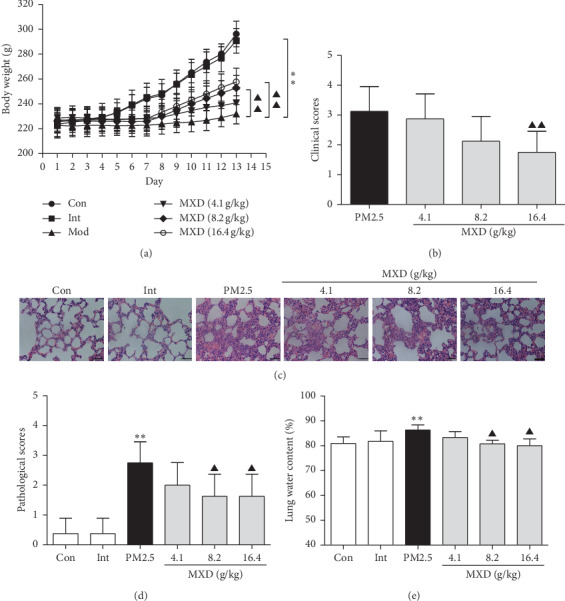
A 7-day continuous treatment with MXD attenuated PM2.5-induced lung injury. (a) Changes in body weight. (b) Clinical score assessment. (c) Representative lung slices of H&E staining, scale bars: 20 *μ*m. (d) Pathological score of lung tissue. (e) Lung water content. Data are shown as mean ± SD, *n* = 8 for body weight assay, clinical score, and lung water content; *n* = 6 for H&E staining and pathological score. ^*∗∗*^*P* < 0.01 vs. the control group; ^▲^*P* < 0.05 and ^▲▲^*P* < 0.01 vs. the PM2.5 group.

In comparison with rats in the control group, rats exposed to PM2.5 alone exhibited more serious weight loss (Figure 1(a), *P* < 0.01) and severe clinical signs (Figure 1(b)), which indicated that PM2.5 instillation resulted in obvious injury. Administration with MXD (16.4 g/kg) significantly promoted weight recovery and improved the clinical signs (*P* < 0.01), preliminarily suggesting that MXD has the potential of mitigating lung injury and promoting weight recovery.

### 3.2. MXD Improved PM2.5-Induced Lung Histopathological Changes

The lung tissue structures and morphological changes were evaluated with H&E staining 2 h after the last drug administration.

As shown in Figure 1(c), the lungs in PM2.5-challenged groups showed classical characteristic of lung injury, including the thickening of alveoli septum, haemorrhage, and edema cavitation, which were obviously improved by MXD (8.2 g/kg and 16.4 g/kg). The quantitative analysis also indicated that PM2.5 elevated the pathological score of lung tissue (*P* < 0.01), and treatment with MXD (8.2 g/kg and 16.4 g/kg) significantly reduced the pathological score (Figure 1(d), *P* < 0.05).

### 3.3. MXD Attenuated Lung Edema after PM2.5 Exposure

As shown in Figure 1(e), PM2.5 instillation significantly increased the water content in the lungs (*P* < 0.01 vs. the control group), indicating the exposure of PM2.5 resulted in serious lung edema. However, treatment with MXD (8.2 g/kg and 16.4 g/kg) decreased the lung water content (*P* < 0.05 vs. the PM2.5 group), suggesting the protection of MXD against lung edema.

### 3.4. MXD Protected Lung Epithelial Barrier Function In Vivo

Except for resulting in lung edema, the disruption of alveolar capillary barrier after serve lung injury may also result in the leakage of hematocytes and plasma proteins into alveolar spaces. Hence, 2 h after the last drug administration, the evaluation of the total cell count and protein concentration in BALF was conducted.

**Figure 2:**
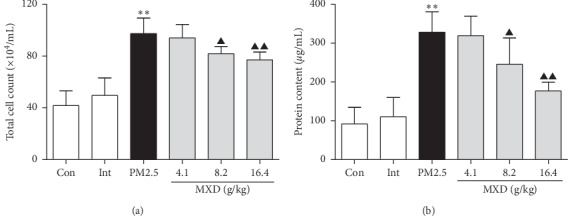
A 7-day continuous treatment with MXD protected lung barrier function. (a) Total cell count in BALF. (b) Protein content in the supernatant of BALF. Data are shown as mean ± SD, *n* = 6. ^*∗∗*^*P* < 0.01 vs. the control group; ^▲^*P* < 0.05 and ^▲▲^*P* < 0.01 vs. the PM2.5 group.

As can be seen in Figures 2(a) and 2(b), the total cell count and protein concentration in BALF of the model group were significantly higher than those of the control group (*P* < 0.01), indicating that PM2.5 challenge contributed to the disruption of alveolar barrier function. Notably, treatment with MXD (8.2 g/kg and 16.4 g/kg) attenuated the leakage of cell and protein into BALF (*P* < 0.05 and *P* < 0.01), suggesting that MXD protected the barrier function of lungs against PM2.5 stimulation.

### 3.5. MXD Inhibited PM2.5-Enhanced Pulmonary EMT in Rats

**Figure 3:**
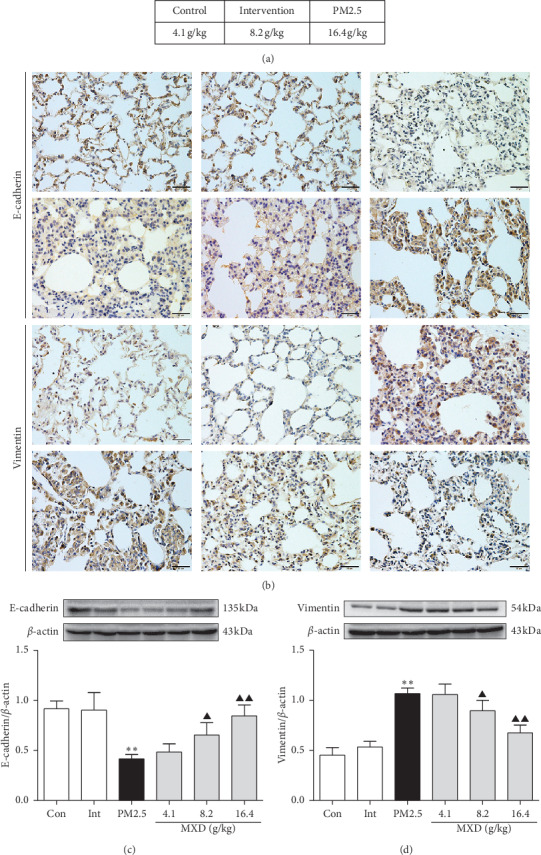
A 7-day continuous treatment with MXD ameliorates EMT in rat lungs. (a) IHC staining of E-cadherin in rat lungs, scale bars: 20 *μ*m. (b) IHC staining of vimentin in rat lungs, scale bars: 20 *μ*m. (c) Protein blots and quantitative analysis of E-cadherin. (d) Protein blots and quantitative analysis of vimentin. Protein expression was expressed as a ratio to endogenous control *β*-actin. Data are expressed as mean ± SD, *n* = 3. ^*∗∗*^*P* < 0.01 vs. the control group; ^▲^*P* < 0.05 and ^▲▲^*P* < 0.05 vs. the PM2.5 group.

The IHC staining assay was first introduced for the detection of E-cadherin, an epithelial marker, and vimentin, a mesenchymal marker. As shown in Figure 3(a), lung tissues from rats exposed to PM2.5 showed fewer E-cadherin positive cells when compared with the control group and intervention group. The reduction in the expression of E-cadherin could be partially reversed by MXD administration. In contrast, the expression of vimentin increased in PM2.5-stimulated rats, which was decreased significantly after MXD intervene (Figure 3(b)).

As shown in Figures 3(c) and 3(d), exposure to PM2.5 significantly downregulated E-cadherin expression (*P* < 0.01) and upregulated that of vimentin (*P* < 0.01), which was reciprocally regulated by MXD at 8.2 g/kg and 16.4 g/kg (*P* < 0.05 and *P* < 0.01). Results derived from western blot were in accordance with the IHC staining results (Figures 3(c) and 3(d)). Western blot results showed that MXD inhibited the PM2.5-initiated EMT in rat lung tissues.

### 3.6. MXD Suppressed PM2.5-Activated TGF-*β*/Smad Signaling and Upregulated the Tight-Junction Expression in Rats

**Figure 4:**
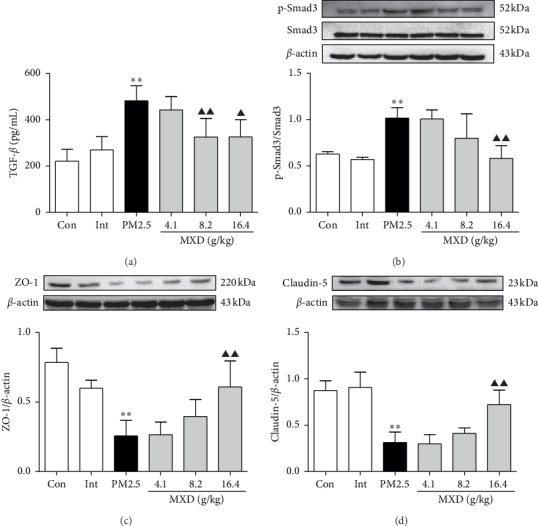
A 7-day continuous treatment with MXD suppressed TGF-*β*/Smad signaling pathway and upregulated the expression of tight junctions. (a) Content of TGF-*β* in the supernatant of lung tissue homogenates. (b) Representative protein bands and quantitative analysis of p-Smad3 in rat lung tissues. (c) Representative protein bands and quantitative analysis of ZO-1. (d) Representative protein bands and quantitative analysis of claudin-5. Data are expressed as mean ± SD, *n* = 3. ^*∗∗*^*P* < 0.01 vs. the control group; ^▲^*P* < 0.05 and ^▲▲^*P* < 0.01 vs. the PM2.5 group.

Considering the TGF-*β*/Smad signal pathway is commonly tangled in fibrotic lung diseases, and we therefore explored whether MXD affected this classical fibrosis-related pathway in rat lung tissues. As can be seen in Figure 4(a), PM2.5 instillation increased the concentration of TGF-*β*1 in lung tissue (*P* < 0.01). Administration of MXD (8.2 g/kg and 16.4 g/kg) significantly inhibited the increase in TGF-*β*1 concentration in lung tissue (*P* < 0.01 and *P* < 0.05). Meanwhile, MXD (8.2 g/kg and 16.4 g/kg) significantly inhibited PM2.5-strengthened phosphorylation of Smad3 (Figure 4(b), *P* < 0.05 and *P* < 0.01), indicating a negative regulation of MXD on TGF-*β*/Smad pathway activation in rat lungs after PM2.5 stimulation. Besides, the expression of ZO-1 and claudin-5 was reduced after PM2.5 stimulation (*P* < 0.01), which was reversed by 16.4 g/kg of MXD (Figures 4(c) and 4(d), *P* < 0.01). Our results indicated the maintenance of MXD on the expression of tight junctions.

### 3.7. Determination of the Optimal Concentration of PM2.5 and MXD-Medicated Serum In Vitro

**Figure 5:**
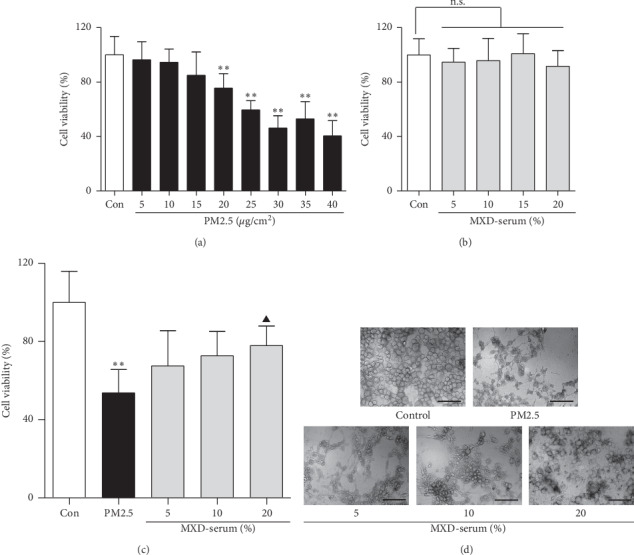
A 48-hour incubation with MXD-medicated serum reduced PM2.5-induced primary alveolar cell injury. (a) Cytotoxic analysis of PM2.5. (b) Optimal concentration selection of MXD-medicated serum. (c) Protective effect of MXD-medicated serum on PM2.5-induced primary alveolar cells injury. (d) MXD-medicated serum improved morphologic changes of primary alveolar cells, scale bars: 60 *μ*m. Data are shown as mean ± SD, *n* = 6. ^*∗*^*P* < 0.05 and ^*∗∗*^*P* < 0.01 vs. the control group; ^▲^*P* < 0.05 vs. the PM2.5 group.

The optimal dose of PM2.5 and MXD for the present study was determined using the CCK8 kits. Primary alveolar cells were continuously exposed to PM2.5 at different concentrations (5, 10, 15, 20, 25, 30, 35, and 40 *μ*g/cm^2^) for 48 h. PM2.5 at the dose of 25 *μ*g/cm^2^ significantly decreased the cell viability by about 50%, which was chosen for the follow-up experiments (Figure 5(a)).

To evaluate the protection of MXD-medicated serum against PM2.5-induced epithelial injury, the nontoxic concentration ranges of medicated serum and the protective effects of different concentrations of medicated serum were examined. As shown in Figure 5(b), compared with the control group, MXD-medicated serum (5%–25%) showed no significant cytotoxicity (*P* > 0.05), indicating medicated serum was nontoxic at the selected concentrations. Accordingly, the three concentrations (5%, 10%, and 20%) were selected for the subsequent experiments.

Furthermore, as shown in Figures 5(c) and 5(d), after PM2.5 stimulation, the cell viability decreased significantly (*P* < 0.01) and the cell shape changed from a cobblestone-like appearance to an elongated spindle-shaped appearance, which was alleviated by incubation with 10% and 20% medicated serum (*P* < 0.05 and *P* < 0.01).

### 3.8. MXD Attenuated TGF-*β*/Smad-Dependent EMT and Upregulated Tight-Junction Protein Expression in Primary Type II Alveolar Cells

**Figure 6:**
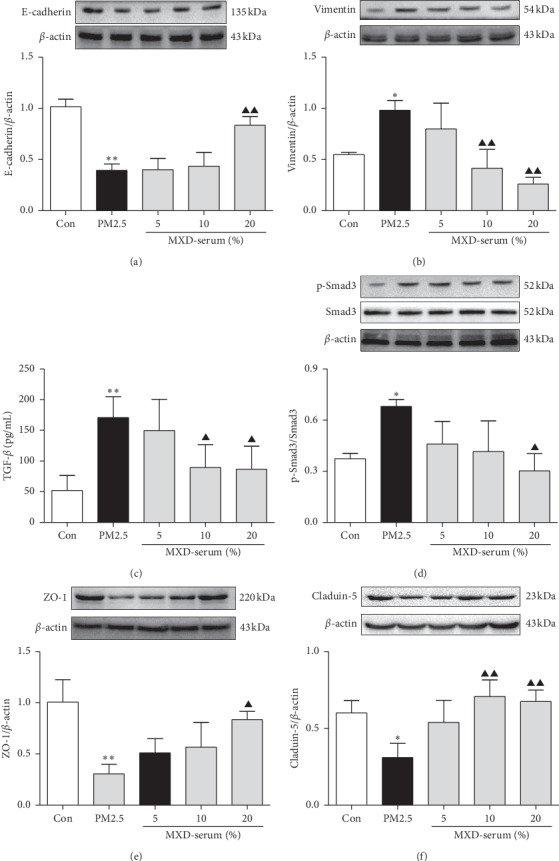
A 48-hour incubation with MXD-medicated serum attenuated TGF-*β*/Smad-dependent EMT and upregulated tight-junction protein expression in primary alveolar cells. (a) Western blot analysis of E-cadherin. (b) Western blot analysis of vimentin. (c) Content of TGF-*β*1 in the culture supernatant of primary alveolar cells. (d) Western blot analysis of p-Smad3. (e) Western blot analysis of ZO-1. (f) Western blot analysis of claudin-5. Data are expressed as mean ± SD, *n* = 3. ^*∗*^*P* < 0.05 and ^*∗∗*^*P* < 0.01 vs. the control group; ^▲^*P* < 0.05 and ^▲▲^*P* < 0.01 vs. the PM2.5 group.

As presented in Figures 6(a) and 6(b), compared with cells in the control group, PM2.5 incubation significantly weakened the expression of E-cadherin (*P* < 0.01) and increased vimentin expression (*P* < 0.01). In accordance with the results observed in rat lung tissues, treatment with 20% MXD-medicated serum reciprocally regulated the expression of E-cadherin and vimentin (*P* < 0.01). As shown in Figure 6(c), 20% MXD-medicated serum significantly inhibited the PM2.5-enhanced release of TGF-*β* (*P* < 0.01). As can be seen in Figure 6(d), PM2.5 enhanced the phosphorylation of Smad3 (*P* < 0.01), and 20% medicated serum inhibited the phosphorylation of Smad3 (Figure 6(d), *P* < 0.05). Figures 6(e)to 6(f) show that PM2.5 significantly downregulated the expressions of ZO-1 (*P* < 0.05) and claudin-5 (*P* < 0.01), which were reversed by 20% medicated serum (*P* < 0.01). These results revealed that MXD-medicated serum suppressed PM2.5-induced EMT, blocked TGF-*β*/Smad pathway, and protected against tight-junction disruption in alveolar epithelial cells.

### 3.9. MXD Exerts Protection against EMT in Primary Alveolar Cells through TGF-*β*/Smad Pathway

**Figure 7:**
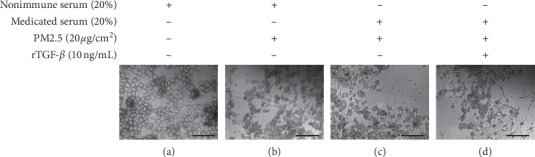
rTGF-*β* inhibited the maintenance of epithelial morphology by MXD-medicated serum after incubation for 48 h. (a) Control group, primary alveolar cells showed a cobblestone-like morphology with clear cell adhesion. (b) PM2.5 group, primary alveolar cells showed a long shuttle shape, and the space between cells was increased. A large number of cells were detached. (c) Treatment group, the alternation in morphological changes was attenuated, and most of the cells showed normal epithelial morphology. (d) rTGF-*β* group, the effect of MXD-medicated serum was alleviated, evidenced by most of the cells exhibited a long shuttle shape, and the space between cells was increased, scale bars: 60 *μ*m (400x).

Results from experiments both in vivo and in vitro indicated that MXD inhibited PM2.5-triggered fibrosis rat lung tissues. Besides, previous studies have shown that PM2.5-induced EMT was TGF-*β* dependent to a certain extent [17, 18, 49–51]. Hence, a verification experiments in vitro was conducted for further exploration of the underlying mechanisms. As shown in Figure 7, the primary alveolar cells treated with nonimmune serum alone showed a cobblestone-like morphology with clear cell adhesion. After stimulation with PM2.5, primary alveolar cells exhibited a long shuttle shape with increased space between cells which was in accordance with our results in Section 3.7. The alternation in morphology was reversed by 20% MXD-medicated serum. In line with expectations, the maintenance of lining epithelium morphology by MXD-medicated serum was abolished by coincubation with rTGF-*β*.

**Figure 8:**
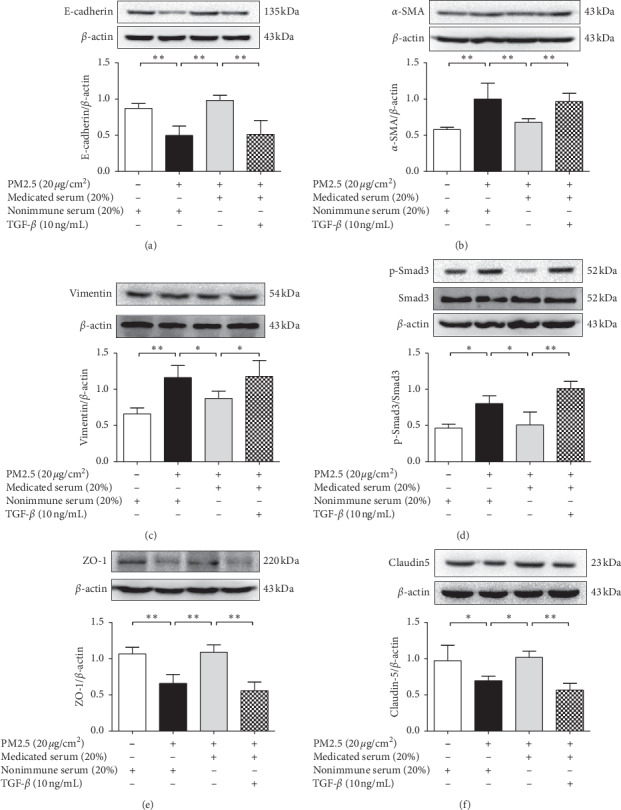
Effect of rTGF-*β* on the regulation of EMT-related proteins and tight-junction proteins after incubation for 48 h. (a) Effect of rTGF-*β* on the expression of E-cadherin. (b) Effect of rTGF-*β* on the expression of *α*-SMA (another fibroblastic marker). (c) Effect of rTGF-*β* on the expression of vimentin. (d) Effect of rTGF-*β* on the phosphorylation of Smad3. (e) Effect of rTGF-*β* on the expression of ZO-1. (f) Effect of rTGF-*β* on the expression of claudin-5. Data are expressed as mean ± SD, *n* = 3. ^*∗*^*P* < 0.05 and ^*∗∗*^*P* < 0.01.

As Figure 8 shows, western blot results revealed that the stimulatory effect of PM2.5 and the therapeutic effect of MXD-medicated serum on the expressions of EMT-related proteins and tight-junction proteins were in accordance with the results above. Notably, coincubation with rTGF-*β* inhibited E-cadherin expression (*P* < 0.01) and restored vimentin expression (*P* < 0.05) and *a*-SMA (another mesenchymal marker, *P* < 0.05) in contrast with the treatment group. Moreover, coincubation with rTGF-*β* also enhanced the phosphorylation of Smad3 (*P* < 0.01) and blocked the effects of MXD-medicated serum in upregulating the expression of ZO-1 (*P* < 0.05) as well as claudin-5 (*P* < 0.01) in comparison with the MXD-medicated serum treatment group.

## 4. Discussion

As a major environmental risk factor, air pollution contributes to human health risks [52]. As is determined by the World Health Organization, two million deaths were caused by respirable PM annually worldwide [19]. The most important pollutant during the hazes is PM2.5, which will be stranded deep in the bronchi upon inhalation, and then the gas exchange in lung will be severely affected [53].

Alveolar epithelial barrier is composed of windpipe surface mucus and fluids, and tight-junction complexes that formed by contribution of surrounding epithelial cells [54]. The upper tight junctions and the adherent junctions below interact with each other to form the functional barrier [55]. The extracellular domain of homophilic E-cadherin interacts with each other to form an intercellular contact, the so-called adherent junction, which contributes to the integrity of barrier function [56]. E-cadherin stabilizes contacts between cells through interacting with *β*-catenin and forming the E-cadherin/*β*-catenin complex. The diminishment in E-cadherin-mediated interactions between cells has been observed during the lung injury in an animal model, contributing to the disruption of epithelial barrier. Except for the underlying adherent junction, the apical tight junctions, mainly composed of ZOs, claudins, and occludin, are also indispensable for the epithelial barrier function. Due to the direct contact with the outside world, the epithelium is more sensitive to experience structural alterations faced with particle challenge.

Clinical and preclinical studies both have shown that ambient PM2.5 mainly does harm to the respiration system and the cardiovascular system [8, 57]. Recent research studies have demonstrated that the critical mechanisms mediating respiratory and cardiovascular injury induced by PM2.5 are mainly associated with inflammatory response and fibrosis [58–60]. PM2.5-induced inflammatory reaction is closely related to the release of inflammatory cytokines including TGF-*β*, IL-1*β*, and TNF-*α*. Among these cytokines, TGF-*β*, an efficient profibrotic cytokine, is one of the most essential factors initiating the phenotypic transition of fibroblasts to myofibroblasts, which mediates the EMT progress [61–63]. The critical role of TGF-*β*-mediating Smad (mainly Smad3) signaling has been demonstrated in EMT associated with PM2.5 stimulation, bleomycin challenge, and tumour progression/development [18, 19, 64–69]. TGF-*β* signals are transduced through transmembrane serine/threonine kinase receptors. The type I receptors will be internalized into endosome after binding with TGF-*β*. Then, the Smad anchor for receptor activation (SARA) mediates formation of complexes with Smad (Smad2 or Smad3), after which Smad will be phosphorylated at serine residues. The phosphorylation results in the interaction between Smad2/3 and Smad4, followed by translocation to the nucleus where they interact with other transcription factors to regulate the transcription of EMT-related genes such as vimentin and *α*-SMA, by interacting with Smad-binding elements [17, 70, 71]. Based on the fact that Smad3 plays a more critical role in TGF-*β*-mediated EMT process, we focused on the phosphorylation level of Smad3 in this research [72–74]. During the EMT process, alterations of morphology in epithelial cells could be observed, evidenced by the diminishment of epithelial cell markers, such as E-cadherin and the acquisition of mesenchymal cell markers (vimentin and *α*-SMA) [63]. With loss of differentiated columnar cells, the barrier function is often compromised [75]. In addition, EMT also promote the degradation of E-cadherin-supported adherent junctions and downregulates the tight-junction expression, further deteriorating the disruption of the epithelial barrier and resulting in fibrosis [76]. Drastic changes in cellular phenotype are observed in oncogenic transformation, metastasis formation, and tumour invasiveness by epithelium-derived tumours. Exposure to PM2.5 can lead to bronchus asthma, pulmonary fibrosis, and even lung carcinoma [77–79].

**Figure 9:**
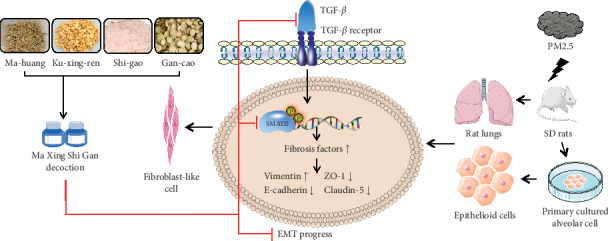
Schematic diagram depicting the proposed mechanism for MXD-mediated protection against PM2.5-induced EMT through inhibiting TGF-*β*/Smad. MXD decreased TGF-*β* expression and downstream Smad3 activation and then reversed the EMT process (evidenced by the upregulation of E-cadherin and the downregulation of vimentin). In addition, the upregulation of tight-junction proteins (ZO-1 and claudin-5) was involved in the protection of MXD. Arrow: stimulatory modification; perpendicular line: inhibitory modification.

In the present research, we observed the protection of MXD against lung injury induced by PM2.5 in rats. Treatment with MXD limited the weight loss, decreased clinical signs, reduced lung edema, and inhibited the disruption of epithelial barrier. Histological analysis further verified that MXD significantly attenuated lung tissue injury in vivo. Moreover, MXD also alleviated the development of EMT in the pulmonary parenchyma following PM2.5 challenge, together with the restored expression of tight junction. A previous study has reported that inhibition of the TGF-*β*/Smad signal pathway inhibits EMT and attenuates the pulmonary fibrosis diseases [80]. Considering TGF-*β*/Smad signaling is implicated in fibrosis-related lung diseases and PM2.5-initiated pulmonary fibrosis is partly TGF-*β* dependent, we hypothesized that MXD prevented lung fibrosis via suppressing TGF-*β*-mediated EMT process and examined the regulatory effect of MXD on the TGF-*β*/Smad signal pathway [81]. Notably, MXD alleviated the content of TGF-*β*1 and attenuated the phosphorylation of Smad3, suggesting the antifibrosis effect of MXD through inhibiting the TGF-*β*/Smad signal pathway. Although the alveolar barrier is composed of endothelial and epithelial cells, the critical role of the epithelium is emphasized attributing to the fact that changes in epithelial permeability alone are sufficient result in pulmonary edema [82]. Hence, we used primary alveolar cells to verify the protection of MXD on EMT progress and epithelial barrier disruption. Our results revealed that MXD-medicated serum reciprocally regulated E-cadherin and vimentin expression and suppressed PM2.5-initiated EMT. In accordance with results in vivo, PM2.5 enhanced the secretion of TGF-*β*1 and the activation of Smad3. In addition, MXD-medicated serum upregulated the ZO-1 and claudin-5 expression, supporting the protective effects of MXD in vitro. As most of the results above are correlative evidence, a verification experiment was designed using rTGF-*β*. The regulatory effect of MXD-medicated serum was abolished by r-TGF*β*, indicating that MXD-medicated serum exerted protection at least partially through inhibiting the TGF-*β*/Smad pathway. We have demonstrated that MXD protected alveolar epithelial cells from PM2.5-triggered EMT, which had not been shown before (Figure 9).

Nevertheless, several limitations must be acknowledged. Except for TGF-*β*/Smad, other signals related to EMT progress including PI3K/Akt and ERK should be studied. TGF-*β* signal-dependent EMT is also under the regulation of other EMT-related transcription regulators (including but not limited to Snail, Twist, and ZEB families of transcription factors). The influence of MXD on these factors requires further study. Besides, a burgeoning system science on the basis of a holistic theory integrated with reductionism which has been utilized to systematically elucidate the synergistic mechanisms underlying combination therapy, especially for exploring the mechanism underlying the efficacy of the traditional Chinese medicine compound, maybe a good choice for the deeper mechanism research of MXD [83–88]. Moreover, in lung tissue, resident macrophage and infiltrated neutrophil play critical roles during lung injury, whose effects in fine particles induced pulmonary injury remain to be further explored.

## 5. Conclusion

In summary, we demonstrated in this research that MXD showed protection against PM2.5-induced lung injury both in vivo and in vitro. The mechanism may be reversing the PM2.5-activated TGF-*β*/Smad3 signal pathway, thereby inhibiting the EMT process and maintaining the expression of tight-junction proteins. These results suggest that MXD shows high potential in the therapy of PM2.5-induced lung injury.

## Figures and Tables

**Table 1:** Information list of the 4 herbs of MXD.

Latin name	Chinese name	Main phytochemical fractions	Pharmacological effects
*Ephedrae herba*	Ma-huang	Alkaloids, Essential oils, Flavonoids, polysaccharides	Antitussive effect, antiasthma, antipyretic analgesic effect, anti-inflammation, antiallergic effect, antitumor, antifibrosis [21–27]

*Armeniacae amarum semen*	Ku-xing-ren	Cyanogenic glycosides, alkaloids, amino acids, vitamins, flavonoids, essential oils	Antitussive effect, anti-inflammation, abirritation, antitumor, antifibrosis, immunoregulation [28–32]

*Glycyrrhizae radix preparata*	Gan-cao	Organic acids, alkaloids, flavonoids, saponins, polysaccharides	Anti-inflammation, liver protection, anti-ischemia, antifibrosis, antioxidation, antivirus activity [33–37]

*Gypsum fibrosum*	Shi-gao	Calcium sulfate, inorganic elements (fe, K, Al, and so on)	Antifebrile action, antiallergic, treating sepsis [38, 39]

**Table 2:** Number of animals for different experimental groups.

	Control	Intervention	PM2.5	MXD (g/kg)			Total
				4.1	8.2	16.4	
Clinical signs	8	8	8	8	8	8	48
Body weight	(8)	(8)	(8)	(8)	(8)	(8)	
Lung edema	(8)	(8)	(8)	(8)	(8)	(8)	
H&E staining	(6)	(6)	(6)	(6)	(6)	(6)	
IHC assay	(6)	(6)	(6)	(6)	(6)	(6)	
ELISA assay	(5)	(5)	(5)	(5)	(5)	(5)	
Western blot	(3)	(3)	(3)	(3)	(3)	(3)	
BALF collection	6	6	6	6	6	6	36
Preparation of medicated serum	5	5	—	—	—	—	10
Primary cell extraction	—	—	—	—	—	—	20
Total							114

## Data Availability

The data used to support the findings of this study are available from the corresponding author upon request.

## Abbreviations

ANOVA: Analysis of variance
BALF: Bronchoalveolar lavage fluid
BCA: Bicinchoninic acid
ELISA: Enzyme-linked immunosorbent assay
EMT: Epithelial-to-mesenchymal transition
H&E: Hematoxylin and eosin
IHC: Immunohistochemical
MXD: Ma Xing Shi Gan decoction
PM2.5: Particulate matter less than 2.5 *μ*m in diameter
PVDF: Polyvinylidene fluoride
RIPA: Radioimmunoprecipitation assay
rTGF-*β*: Recombinant transforming growth factor beta
ZOs: Zonula occludens.

## Supplementary Materials

Supplementary Materials Supplementary Figure 1: the safety of MXD was evaluated in mice and rats. Supplementary Figure 2: the successful establishment of lung fibrosis was evidenced by the Masson staining and the expression of Collagen I and III. Supplementary Figure 3: the primary alveolar type II cell was identified by immunocytochemistry staining of pulmonary surfactant-associated protein.
