# Supplementary material for: Ma Xing Shi Gan Decoction Protects against PM2.5-Induced Lung Injury through Suppression of Epithelial-to-Mesenchymal Transition (EMT) and Epithelial Barrier Disruption
Source: Evid Based Complement Alternat Med. 2020 Jun 17;2020:7176589. doi: 10.1155/2020/7176589 (PMC7317335; doi:10.1155/2020/7176589)
Supplement: Supplementary Materials — Supplementary Figure 1: the safety of MXD was evaluated in mice and rats. Supplementary Figure 2: the successful establishment of lung fibrosis was evidenced by the Masson staining and the expression of Collagen I and III. Supplementary Figure 3: the primary alveolar type II cell was identified by immunocytochemistry staining of pulmonary surfactant-associated protein. [file 7176589.f1.pdf]

Ma Xing Shi Gan Decoction protects against PM2.5 induced lung injury through suppression of epithelial-to-mesenchymal transition (EMT) and epithelial barrier disruption

Ye-fang Wang[1‡], Yu-xiang Fei[2‡], Bo Zhao[2], Qi-yang Yin[2], Jian-ping Zhu[2], Guang-hui Ren[2], Bo-wen Wang[2], Wei-rong Fang[2]*, Yun-man Li[2]*

[1] Department of paediatrics, Nanjing Integrated Traditional Chinese and Western Medicine Hospital, Nanjing 210014, PR China
[2] State Key Laboratory of Natural Medicines, School of Basic Medical Sciences and Clinical Pharmacy, China Pharmaceutical University, Nanjing 210009, PR China
[‡]These two authors contributed equally to this work.

Correspondence should be addressed to Wei-rong Fang; weirongfang@163.com and Yun-man Li; yunmanlicpu@163.com

Supplementary Materials:

1. Methods

1.1. Oral toxicity assay of Ma Xing Shi Gan Decoction (MXD) in mice

SPF ICR mice (23±2 g, half male and half female) purchased from Comparative Medical Centre of Yangzhou University were randomly divided into control group and intervention group. Mice in intervention group were orally administered with MXD at 40 g/kg/day (0.2 mL/10 g body weight), which was about 30 times of the dosage in clinical application. Mice in control group were administered with the same volume of normal saline. The drug was administered for 7 consecutive days. Mice were weighed daily to evaluated the effect of drugs on body weight. After the experimental period (7 days), mice were starved for 24 h. Then, rats were sacrificed and the organs including heart, liver, spleen, lung, kidney and brain were removed from sacrificed mice. All organs were washed in normal saline, sucked dry, weighted and observed with the naked eye. Organ coefficient was calculated as follows:

$$\text{Organ coefficient} = \frac{\text{The wet weight of respective organ (g)}}{\text{Body weight (g)}}$$

1.2. Oral toxicity assay of MXD in rats

The rats in control group and intervention group (16.4 g/kg) used for H&E staining in the main body of the text were also used for the toxicity assay. The fixed tissues (heart, liver, spleen, kidney, brain) were sliced into 5-μm sections and then H&E staining was conducted. Sections were observed using a light microscope (Olympus, Japan, BX53) by a researcher blinded to the grouping.

1.3 Masson staining

To verify the successful establishment of lung fibrosis, lung collagen deposition was determined by Masson staining as described previously with slight modification [1]. The same batch of fixed lung tissues were used for H&E staining and Masson staining. Sections (4 μm) were prepared and stained with Masson's trichrome. Briefly, paraffin sections were dewaxed, followed by washing with distilled water. Sections were then stained with Weigert hematoxylin for 10 min for nuclei identification. After fully washing with distilled water, slides were incubated with Ponceau Masson Acid fuchsin solution for 10 min. Sections were then briefly immersed in a 0.5% aqueous acetic acid solution for 15 s and incubated with 1% phosphomolybdic acid for 5 min, followed by direct staining with aniline blue or light green liquid dye for 5 min. After an immersion in 1% acetic acid for 1 min, sections were dehydrated with 95% ethanol

(5 min×2) and 100% ethanol (5 min×2), and transparent xylene (5 min×2) and then cemented with neutral gum. Sections were observed using a light microscope (Olympus, Japan, BX53) by a researcher blinded to the grouping.

1.4 Western blot

The expression of collagen I and collagen III were quantified with specific rabbit polyclonal antibodies: anti-collagen I (Wanleibio, Shenyang, China; dilution 1:800) and anti-collagen III (Wanleibio, Shenyang, China; dilution 1:800). Western blot was conducted as described in section 2.17.

1.5 Identification of primary alveolar type II cell

Primary alveolar type II cell was identified by immunocytochemistry against pulmonary-surfactant associated protein (SPA), the marker of alveolar type II cell. Briefly, isolated primary alveolar type II cell were seeded onto a cell slide at 5×10^4/slide and cultured for 24 h, after which cells were washed twice with PBS, fixed with 4% paraformaldehyde for 30 min and permeabilized with 0.1% Triton X-100 in PBS for 10 min. Slides were then incubated with 3% hydrogen peroxide solution at room temperature for 30 min followed by 30 min with normal goat serum to block endogenous peroxidase and non-specific staining. Cells were then incubated at room temperature for 2 h with anti-SPA (Abbkine, Wuhan, China). Slides were washed twice with PBS and then incubated with Goat Anti-Rabbit IgG HRP (Abways, Shanghai, China, dilution 1:500) at room temperature for 30 min. A peroxide reaction using diaminobenzidine as a substrate was performed. Slides were counterstained with hematoxylin and observed under a light microscope (Olympus, Japan, BX53).

1.6 Statistical analysis

Data were visualized using GraphPad Prism 5.0. All statistical analyses were performed using SPSS 19.0 software and analysed by One-way analysis of variance (ANOVA) followed by LSD post hoc test (variance homogeneity) to compare the difference between groups. When the variance was heterogeneous, Welch’s ANOVA would be introduced followed by Games Howell test. A value of $P<0.05$ was considered as statistically significant.

2.Results

2.1 MXD showed no obviously toxic side effect in mice and rats

During the period of drug delivery, no whole-body reaction or death was observed. As shown in Supplementary FIGURE 1(a) to (c). Compared with control group, no significant changes in body weight, organ coefficient or morphology of main organs

were observed in intervention group, preliminarily indicating administration with MXD for 7 consecutive days showed no apparent toxicity in mice.

As shown in Supplementary FIGURE 1(d), H&E staining of rat tissue samples showed that there was no evident pathological change nor inflammatory cell infiltration in tissue sections, indicating the satisfying safety of MXD in this study.

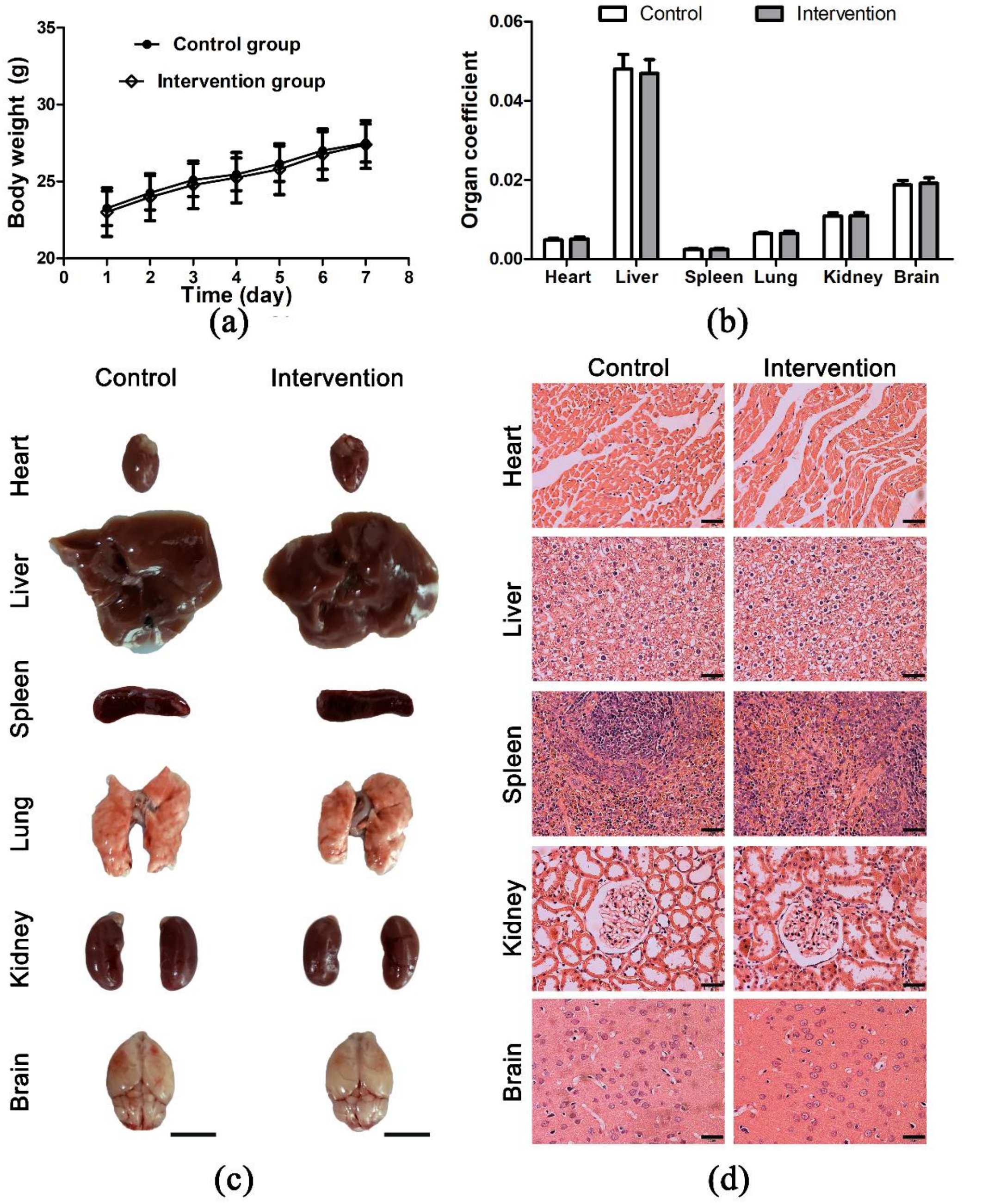

Supplementary FIGURE 1. MXD showed no obvious toxicity in mice and rats. (a) Body weight changes in mice (n=10). (b) Mice organ coefficient (n=10). (c) Examination of

main organs in mice with naked eyes (Scale bar=1 cm). (d) H&E staining sections of main organs in rats (Scale bar, 20 μm). Data are shown as mean ± SD.

2.2 MXD inhibited the collagen deposition in lung tissue

As shown in Supplementary FIGURE 2 (a), Masson staining revealed that marked lung fibrosis was induced after PM2.5 exposure. Compared with the control group, the collagen deposition (blue areas) was significantly higher in PM2.5 group. In comparison with PM2.5 group, administration with MXD significantly attenuated the accumulation of collagen fibre in lung tissues.

Moreover, as shown in Supplementary FIGURE 2 (b) to (c), compared with control group, the lung fibrosis was further evidenced by the increased expression of collagen I and collagen III in PM2.5 group. Administration with MXD at 16.4 g/kg significantly downregulated the expression of collagen I and collagen III ($P<0.05$ and $P<0.01$), indicating the anti-fibrotic benefit of MXD.

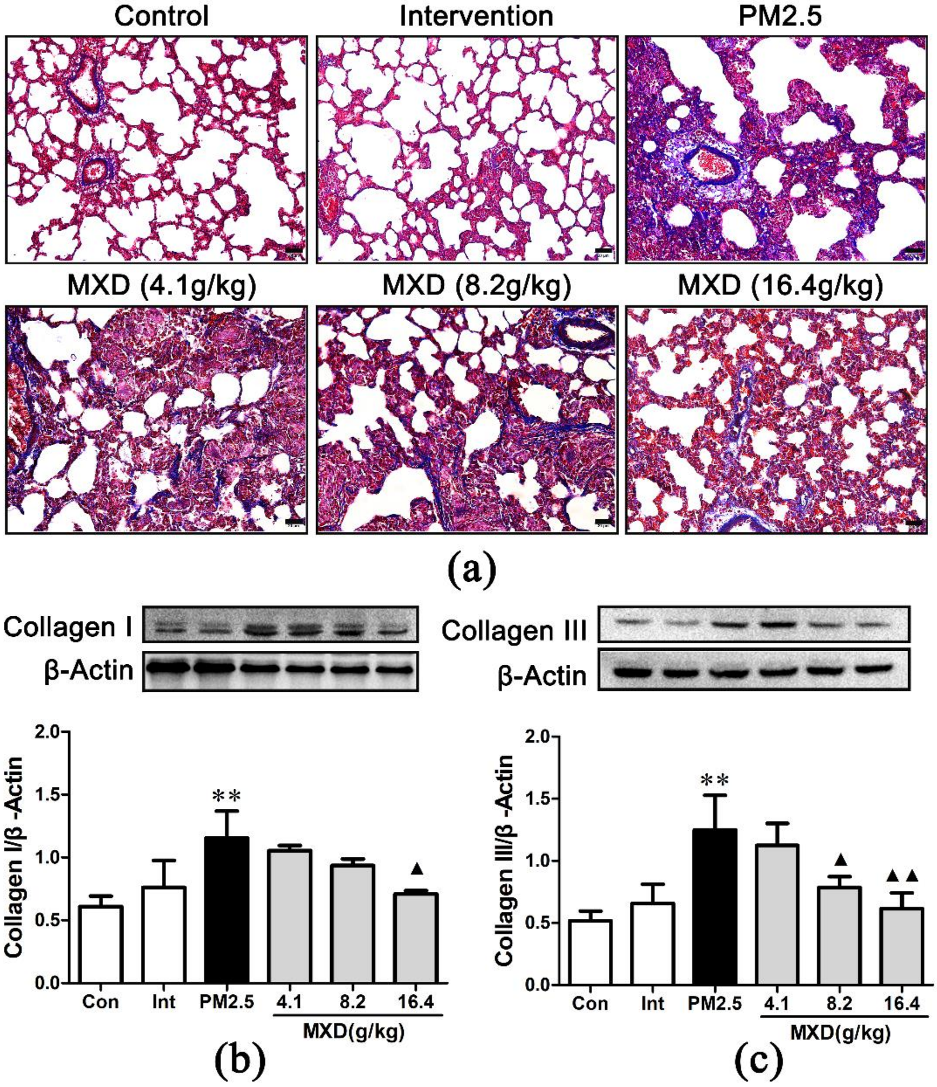

Supplementary FIGURE 2. MXD inhibited PM2.5-induced collagen deposition and the expression of collagen I and III in rat lungs. (a) Masson staining in rat lungs, scale bar= 20 μm. The expression of collagen I (b) and collagen III (c) in lungs. Data are shown as mean ± SD. ***P*<0.01 vs. control group; ▲*P*<0.05, ▲▲*P*<0.01 vs. PM2.5 group.

2.3 Primary alveolar type II cell was identified by SPA expression

As shown in Supplementary FIGURE 3, majority of isolated cells showed SPA positive immunoreaction, indicating the purity of primary alveolar type II cell basically meet the design requirements.

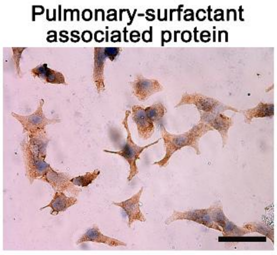

Supplementary FIGURE 3. Immunocytochemistry staining of SPA in primary alveolar type II cell (Scale bar=30 μm)

References

[1] Y. Jing, H. Zhang, Z. Cai, Y. Zhao, Y. Wu, X. Zheng, Y. Liu, Y. Qin, M. Gu, J. Jin, Bufei huoxue capsule attenuates PM2. 5-induced pulmonary inflammation in mice, Evidence-Based Complementary and Alternative Medicine 2017 (**2017**).
